# Corrigendum: Exploring the Niche Concept in a Simple Metaorganism

**DOI:** 10.3389/fmicb.2020.633429

**Published:** 2021-01-12

**Authors:** Peter Deines, Katrin Hammerschmidt, Thomas C. G. Bosch

**Affiliations:** ^1^Zoological Institute, Christian Albrechts University Kiel, Kiel, Germany; ^2^Institute of General Microbiology, Christian Albrechts University Kiel, Kiel, Germany

**Keywords:** fundamental niche, realized niche, microbiome, species abundance, microbial traits, *Hydra*

In the published article, there was an error regarding the affiliations for Peter Deines. As well as having affiliation 1, he should also have RD3 Marine Symbioses, GEOMAR Helmholtz Centre for Ocean Research Kiel, Kiel, Germany as a present address.

The authors apologize for this error and state that this does not change the scientific conclusions of the article in any way. The original article has been updated.

